# Isolated cell-bound membrane vesicles (CBMVs) as a novel class of drug nanocarriers

**DOI:** 10.1186/s12951-020-00625-2

**Published:** 2020-05-06

**Authors:** Yang Zhang, Yang Liu, Wendiao Zhang, Qisheng Tang, Yun Zhou, Yuanfang Li, Tong Rong, Huaying Wang, Yong Chen

**Affiliations:** 1 Jiangxi Key Laboratory for Microscale Interdisciplinary Study, Institute for Advanced Study, Nanchang University, 999 Xuefu Ave., Honggutan District, Nanchang, Jiangxi 330031 People’s Republic of China; 2 School of Materials Science and Engineering, Nanchang University, Nanchang, Jiangxi 330031 People’s Republic of China

**Keywords:** Cell-bound membrane vesicles (CBMVs), Drug delivery systems, Doxorubicin (Dox), Extracellular vesicles (EVs), Tumors

## Abstract

**Background:**

Cell-bound membrane vesicles (CBMVs) are a type of membrane vesicles different from the well-known extracellular vesicles (EVs). In recent years, the applications of EVs as drug delivery systems have been studied widely. A question may arise whether isolated CBMVs also have the possibility of being recruited as a drug delivery system or nanocarrier?

**Methods:**

To test the possibility, CBMVs were isolated/purified from the surfaces of cultured endothelial cells, loaded with a putative antitumor drug doxorubicin (Dox), and characterized. Subsequently, cellular experiments and animal experiments using mouse models were performed to determine the in vitro and in vivo antitumor effects of Dox-loaded CBMVs (Dox-CBMVs or Dox@CBMVs), respectively.

**Results:**

Both Dox-free and Dox-loaded CBMVs were globular-shaped and nanometer-sized with an average diameter of ~ 300–400 nm. Dox-CBMVs could be internalized by cells and could kill multiple types of cancer cells. The in vivo antitumor ability of Dox-CBMVs also was confirmed. Moreover, Quantifications of blood cells (white blood cells and platelets) and specific enzymes (aspartate aminotransferase and creatine kinase isoenzymes) showed that Dox-CBMVs had lower side effects compared with free Dox.

**Conclusions:**

The data show that the CBMV-entrapped Doxorubicin has the antitumor efficacy with lower side effects. This study provides evidence supporting the possibility of isolated cell-bound membrane vesicles as a novel drug nanocarrier.

## Background

For more than a decade, extracellular vesicles (EVs) have been a research frontier in cell biology due to the long-range cell-to-cell communication functions and in medicine due to the correlation with multiple diseases [1–4]. Different types of EVs are characterized due to different properties in size, structure, surface marker, biogenesis, cargo, uptake, among others [3–5], including exosome (generally < 100 nm in diameter), microvesicle or microparticle (~ 0.1–1 µm), and apoptotic body (~ 1–5 μm) [3–5]. In recent years, EVs (particularly exosome) have been widely applied as efficient drug delivery systems [6–9].

Cell-bound membrane vesicles (abbreviated as CBMVs in this paper) are the membrane vesicles with a size of hundreds of nanometers (up to ~ 1 μm) existing on the surfaces of many cells. These vesicles have long been regarded as the precursors of EVs (particularly microvesicles due to similar size and shape) prior to the release from cell surfaces [10–12]. Recently, however, we have excluded the possibility of CBMVs being the precursors of the well-known types of EVs. First, the fluorescence detection showed that CBMVs are not co-localized with the major surface markers (e.g., CD63, CD31, CD62E, LAMP-1, phosphatidylserine, etc.) of EVs [13]; second, it was found that CBMVs are resistant to detergents (e.g., Triton X-100, sodium dodecyl sulfate or SDS, etc.) whereas there are no previous reports supporting that EVs (particularly microvesicles) are detergent-resistant as an entity [13]; third, dynamic single-vesicle tracking of CBMVs on the surfaces of individual living cells did not find the release of CBMVs [14]; forth, in situ topographical imaging of the actin cytoskeleton under individual CBMVs [15] excludes the possibility of CBMVs being exosomes.

**Scheme 1:**
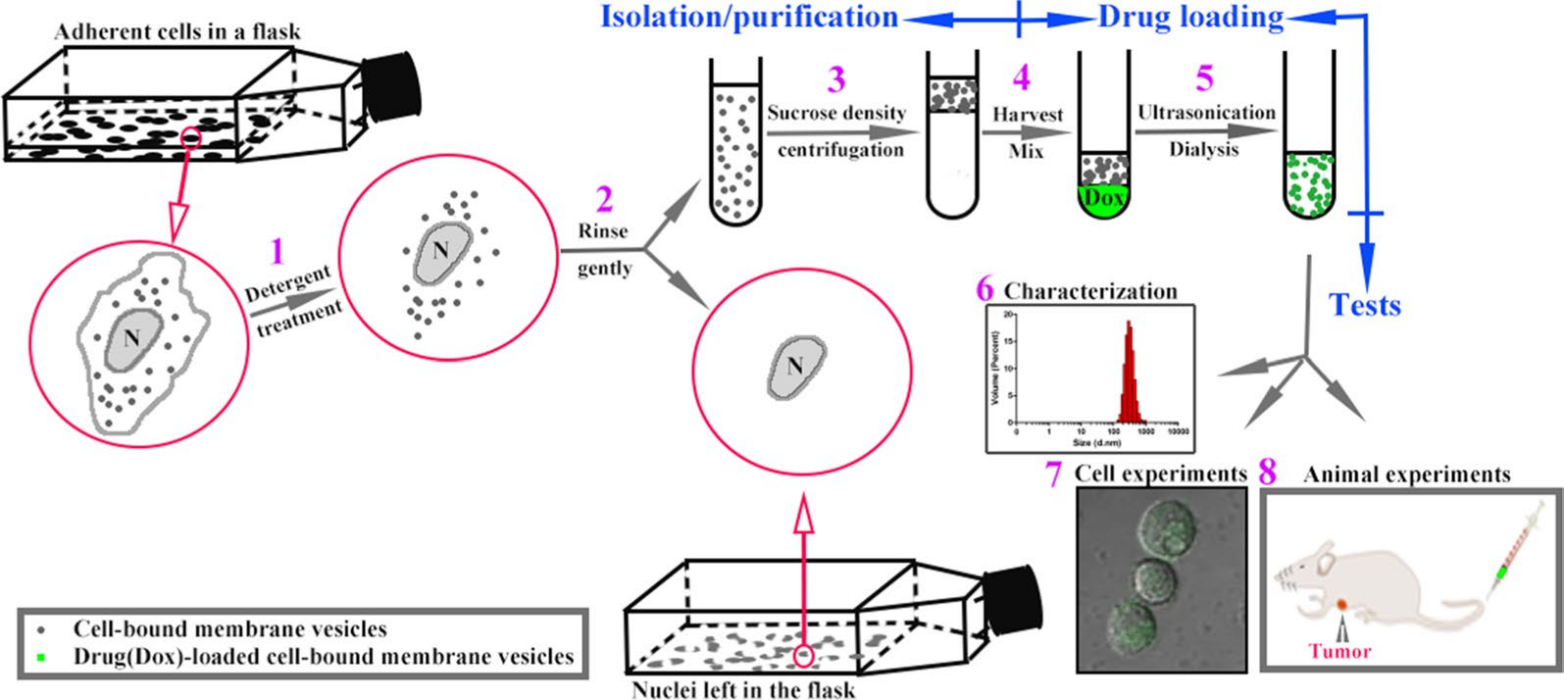
Schematic diagram briefly shows the isolation (steps 1 and 2) and purification (step 3) of cell-bound membrane vesicles (CBMVs) from adherent cells, drug (doxorubicin) loading (steps 4 and 5), characterization (step 6), and evaluation of drug efficacy at cellular (step 7) and animal (step 8) levels

Being inspired by the numerous successful reports of extracellular vesicles (EVs) as drug delivery systems, we hypothesized that cell-bound membrane vesicles (CBMVs) also can be recruited as a drug delivery system if individual CBMVs are able to be isolated from the cell surfaces. To separate individual CBMVs from cells, the detergent-resistant property of CBMVs was utilized. To test the abovementioned hypothesis, doxorubicin (a widely used, highly effective antitumor drug) was recruited as a drug model to be carried by CBMVs since doxorubicin is autofluorescent and has been studied as an antitumor drug for other delivery systems including liposomes and extracellular vesicles [16–18]. In this study, the isolation, purification, and drug loading of CBMVs were performed via several simple steps (steps 1–5 in Scheme 1) following with the characterization and drug efficacy verification of Dox-loaded CBMVs (Dox-CBMVS or Dox@CBMVs; steps 6–8 in Scheme 1).

## Methods

### Cells and cell culture

Human umbilical vein endothelial cells (HUVECs), human liver cancer cell line SMMC-7721, and mouse lung cancer cell line LLC Lewis were purchased from the Cell Bank of the Chinese Academy of Sciences (Shanghai, China). Mouse colon cancer cell line CT26.WT and mouse liver cancer cell line Hepa1-6 were purchased from Shanghai Zhong Qiao Xin Zhou Biotechnology Co., Ltd. (Shanghai, China). HUVEC, CT26.WT, and SMMC-7721 cells were routinely cultured in RPMI 1640 medium (Sigma) whereas LLC Lewis and Hepa1-6 cells were cultured in Dulbecco’s Modified Eagle’s medium (DMEM; Sigma). Each medium was supplemented with 10% (w/v) fetal bovine serum (FBS; Biological Industries, Israel) and penicillin–streptomycin solution (Solarbio Science & Technology Co., Shanghai, China) containing 100 U/mL penicillin and 100 μg/mL streptomycin. All cells were used at passage ~ 5.

### Isolation/purification and quantification of cell-bound membrane vesicles

Cell-bound membrane vesicles (CBMVs) were isolated from cultured endothelial cells (HUVECs). After taking from a 5% CO_2_ incubator, HUVECs were rinsed gently three times with warmed-up phosphate-buffered saline (PBS) and treated with 0.1% Triton X-100 at 37 °C for approximately 2–3 min. After removing the detergent and washing twice with PBS, the cells were gently rinsed 1–3 times with PBS to collect the isolated CBMVs into fresh tubes. After spinning at 4 °C first at 1000×*g* for 5 min and then at 10,000×*g* for 30 min to remove the possible objects with relatively large sizes in the pellets (e.g., cell debris, cell nuclei detached from the substrate, vesicle aggregates, etc.), 10% sucrose density centrifugation was performed at 200,000×*g* for 90 min at 4 °C to obtain the vesicle-containing upper layer. LB30 Latex beads (Sigma) and flow cytometry (BD FACSCalibur; BD Biosciences, San Jose, CA) were utilized to quantify the concentration of isolated CBMVs. The samples were divided into three groups: (a) no beads (PBS only); (b) LB30 beads with a known number (e.g. 1.35 × 10^7^ beads in PBS); and (c) CBMVs plus LB30 beads with the same number. The distribution of the particles in the solution were detected by flow cytometry. The number of CBMVs was calculated according to the following equation: N_CBMV_ = N_LB30_ × (P_CBMV_/P_LB30_), where N_CBMV_ and N_LB30_ represent the number of CBMVs and LB30 beads (e.g. N_LB30_ = 1.35 × 10^7^), respectively and P_CBMV_ and P_LB30_ are the percentages of vesicles and beads, respectively.

### Drug loading of isolated cell-bound membrane vesicles

To load the drug, the harvested vesicle-containing solution and 2 mg/mL doxorubicin hydrochloride (abbreviated as doxorubicin or Dox; Kaiji Biotechnology Co., Beijing, China) were mixed (1:1 in volume), ultrasonicated at a 20% power setting by an ultrasonic processor (JY96-II, Ningbo Xinyi Ultrasonic Equipment Co., Ltd., Ningbo, China) using pulsed ultrasound for 6 cycles containing a 30 s “on”, a 30 s “off”, and a 2 min cooling per cycle, and incubated at 37 °C for 1 h to allow for recovery of the vesicle membrane [19]. After dialyzing via cellulose ester dialysis membranes with a 10 k molecular weight cut-off (Solarbio Science & Technology Co., Shanghai, China) to remove free doxorubicin, the samples were stored at 4 °C for other experiments.

### Verification of cell-bound membrane vesicles loaded with or without doxorubicin

To verify the efficacy of the isolation method, the same cells before and after Triton X-100 treatment and after washing for various times were observed by LSM710 confocal microscope (Carl Zeiss, Oberkochen, Germany). The isolated, Dox-loaded vesicles were fluorescently imaged by the confocal microscopy (excitation wavelength at 488 nm). Transmission electron microscopy (JEOL JEM-2100 TEM, Japan) was utilized to visualize the vesicles loaded with or without doxorubicin pre-stained with 1% (w/v) phosphotungstic acid solution (Sinopharm Chemical Reagent Co., Ltd., Shanghai, China).

### Quantification of mean size, zeta potential, and polydispersity index (PDI)

The mean size, zeta potential, and polydispersity index (PDI) of isolated cell-bound membrane vesicles loaded with or without doxorubicin were quantified by dynamic light scattering (DLS) Analyzer (Zetasizer nano zs90, Malvern, UK) as reported in our previous study [20].

### HPLC and quantification of entrapment efficiency (EE) and drug loading efficiency (DL)

High-performance liquid chromatography (HPLC) was used to measure the amount of doxorubicin. A Waters chromatographic system (Waters Technologies, USA) was recruited and the chromatographic separation was performed on a Kinetex C_18_ column (4.6 × 100 mm, 2.6 μm particle size; Phenomenex, USA) at 35 °C (mobile phase: acetonitrile and water (32: 68, v/v) at pH 2.6 by adjusting with 85% orthophosphoric acid; flow rate: 1 mL/min; excitation and emission wavelengths: 475 nm and 555 nm, respectively; daunorubicin hydrochloride, from Solarbio Science & Technology Co. (Shanghai, China), was used as an internal standard). The EE and DL were calculated as the following equations: EE (%) = *W/W*_*t*_ × 100% and DL (%) = *Q/Q*_*t*_ × 100%, where *W* and *Q* are the amount of drug (Dox) loaded in vesicles, whereas *W*_*t*_ and *Q*_*t*_ are the total amount of the feeding doxorubicin and the feeding materials (Dox, vesicles, etc.), respectively.

### Cell viability

3-(4,5-dimethyl-2-thiazolyl)-2,5-diphenyl-2-H-tetrazolium bromide (MTT) assay was used to test the effect of doxorubicin on the viability of multiple cell types including HUVECs, CT26.WT, SMMC-7721, LLC Lewis, and HEPA1-6 cells. Approximately 1 × 10^4^ cells were placed in each well of a 96-well plate and incubated at 37 °C for around 1 day in the 5% CO_2_ incubator. After washing with PBS, Dox-loaded vesicles (PBS, vesicles without doxorubicin, and free doxorubicin were used as controls; the concentration of doxorubicin was 10 μg/mL) were added and incubated with cells at 37 °C for 24 h. After washing twice with PBS, 20 μL of 5 mg/mL MTT (Solarbio Science & Technology Co., Shanghai, China) and 100 μL of fresh medium were added into each well to treat the cells for 4 h. After removing the solution, 100 μL of dimethyl sulfoxide (DMSO; Solarbio Science & Technology Co., Shanghai, China) was added to treat the cells at 37 °C for 10 min. A microplate reader (Rayto) was utilized to measure the absorbance (optical density; OD value) at 570 nm.

### Verification of cellular binding/internalization of dox-loaded vesicles

To determine the binding of Dox-loaded vesicles onto cell surfaces, HUVECs were incubated with Dox-loaded vesicles (10 μg/mL) at 37 °C in a 5% CO_2_ incubator for approximately 1 h, washed three times with PBS, fixed with 4% paraformaldehyde (Xilong Science Co., Ltd., Shantou, China) for 15 min, washed again, and subjected to LSM710 confocal microscope. To determine the internalization of Dox-loaded vesicles, CT26.WT cells were incubated with free doxorubicin (10 μg/mL) or Dox-loaded vesicles (10 μg/mL) at 37 °C for 2 h, washed three times with PBS, fixed with 4% paraformaldehyde for 15 min, washed again, and subjected to LSM710 confocal microscope or BD FACSCalibur flow cytometry (BD Biosciences, San Jose, CA).

To further confirm the binding of Dox-loaded vesicles onto cell surfaces, duel colors were used to display the drug Dox (self-fluorescence) and the vesicles fluorescently stained by Dio (a fluorescent dye for cell membrane; Jiangsu Kaiji Biotechnology Co., Ltd., Nanjing, China), respectively. The isolated CBMVs were incubated with 5 μM Dio at 37 °C for 15 min. After the removal of excess Dio via dialysis, the Dio-stained CBMVs were obtained. The Dox loading of the Dio-stained CBMVs were performed as mentioned above. The Dox-loaded, Dio-stained CBMVs were deposited on a glass coverslip and fluorescently imaged by confocal microscopy. Then, HUVECs were incubated with free Dox, Dio-stained CBMVs loaded without Dox, and Dio-stained CBMVs loaded with Dox, respectively at 37 °C for approximately 1 h, washed three times with PBS, fixed with 4% paraformaldehyde for 15 min, washed again, and subjected to LSM710 confocal microscope. The excitation wavelength for both Dox and Dio was 488 nm. The emission wavelengths for Dox and Dio were 575–585 nm and ~ 501 nm, respectively (in the figure, the blue for Dio is a pseudocolor in order to distinguish the color of Dio from the color of Dox).

### In Vitro drug release assay

The in vitro drug release profile of doxorubicin from vesicles was quantified using the dialysis method as reported in our previous study [20]. A dialysis bag with a molecular weight cutoff of 10 kDa (Solarbio Science & Technology Co., Shanghai, China) containing 1 mL samples were incubated in 200 mL release buffer (PBS; pH 7.4) at 25 °C for 24 h (the buffer was gently stirred on a magnetic stirrer). Approximately 0.5 mL of release buffer was taken (0.5 mL of fresh buffer was supplemented concurrently) at each time point (0.25, 0.5, 1, 2, 3, 4, 6, 8, 12, and 24 h, respectively) and immediately measured via HPLC to determine the concentration of released doxorubicin.

### Animals

Five-week-old male BALB/c mice were purchased from Hunan Slake Jingda Experimental Animals Co., Ltd. (Changsha, China). Ethics approval for the study was obtained from the Nanchang University Health Research Ethics Board and all animal experiments were performed in full compliance with the National Institute of Health Guide for the Care and Use of Laboratory Animals.

### Mouse colon and lung tumor models

For syngeneic CT26 colon tumor model, BALB/c mice were engrafted subcutaneously with approximately 6 × 10^6^ CT26.WT cells in 100 μL PBS and fed for 28 days (the tumor formation rate was 100%) as previously reported [21]. For lung cancer mouse model, approximately 6 × 10^6^ LLC Lewis cells were injected subcutaneously into BALB/c mice (the tumor formation rate was ~ 20%). Around 3 weeks later, tumors were taken from the tumor-bearing mice and cut into small pieces/blocks, and then the tumor blocks with a similar size were engrafted subcutaneously into new batches of mice and the mice were fed for 28 days (the tumor formation rate was 100%).

### Tissue distribution of doxorubicin in tumor-bearing mice

After colon tumor formation, the mice were injected intravenously (via tail vein) with free doxorubicin or doxorubicin-loaded vesicles at a doxorubicin dose of 4 mg/kg, as well as with PBS solutions as a blank control. At 0.5 h and 4 h after a single drug administration, the heart, liver, lung, spleen, kidney, and tumors were taken, weighted, cut into small pieces, and mixed with daunorubicin (3 μg/g tissue). After homogenizing thoroughly in 1 mL of the mobile phase for chromatographic separation, placing on ice for 10 min and spinning at 14,000×*g* for 5 min, the supernatants were subjected to HPLC for the measurement of doxorubicin concentration (the data for the blank control was not showed in the graph because no doxorubicin was detected by HPLC in the blank control group).

### Drug treatments and in vivo determination of drug effects on tumor formation

After the colon cancer cells or lung tumor blocks were engrafted in mice and the tumor volume reached around 100 mm^3^ (~ 1 week later), the mice were randomly divided into 4 groups (PBS group, vesicle group, free doxorubicin group, and the group of doxorubicin-loaded vesicles, respectively; n = 5 mice per group), and the drugs were administrated intravenously every other day (at a doxorubicin dose of 4 mg/kg for Dox-containing groups; ~ 1.56 × 10^6^ CBMVs per injection for vesicle group; via tail vein injection) for 20 days (10 injections for each mouse). The mice were weighted and images every 2–3 days, based on which the dynamic changes in mouse weight and tumor volume were obtained. The tumor volume was calculated as W^2^ × L × 0.5 where W and L represent the width and length of a tumor, respectively as described previously [21]. At the end of animal experiments, after taking photos of the mice, the tumors were taken out from mouse bodies, weighted, imaged, and subjected to other experiments (e.g. histological analysis).

### Histological analysis of tumor and other tissues

At the end of animal experiments, the tissues including tumor, heart, liver, spleen, lung, and kidney were excised from the mice, fixed in 4% paraformaldehyde at room temperature, gradually dehydrated by successively immerging the specimens in 70% (overnight), 80% (~ 4 h), 90% (~ 1 h), and 100% (~ 1 h) ethanol, embedded in paraffin, and sectioned to slides with a thickness of 5 μm. After deparaffinizing with xylene (Tianjin Damao Chemical Reagent Factory, Tianjin, China), the tissue slides were hydrated with ethanol and water, stained with hematoxylin and eosin (i.e. H&E staining; both were from Solarbio Science & Technology Co.), dehydrated, mounted on glass cover slides, and images by an inverted microscope (Nikon LH-M100CB, Japan).

### Apoptotic cell detection in tumor via TUNEL assay

An one step TUNEL apoptosis in situ assay kit was purchased from Jiangsu Kaiji Biotechnology Co., Ltd. (Nanjing, China). The experiment was performed according to the manufacturer’s instruction. Briefly, the tissue slides with a thickness of 5 μm were treated with 1% Triton X-100 for 5 min, washed with PBS for three times, incubated with 100 μL proteinase K solution at 37 °C for 30 min, and washed three times with PBS. After drying, the tissue slides were incubated with 50 μL terminal deoxyribonucleotidyl transferase (TdT) reaction solution at 37 °C in dark for 30 min, washed with PBS for three times, reacted with 50 μL streptavidin-TRITC solution at 37 °C in dark for 30 min, washed with PBS, and stained with DAPI (Solarbio Science & Technology Co., Ltd., Shanghai, China) at room temperature for 10 min. After washing with PBS and sealing up with mounting medium (glycerol: PBS = 6: 4), the tissue slides were subjected to a fluorescence microscopy. The excitation/emission wavelengths of TRITC and DAPI were 543 nm/571 nm and 358 nm/461 nm, respectively.

### Quantification of white blood cells, platelets, aspartate aminotransferase, and creatine kinase isoenzymes

At the end of the treatments, mouse blood was collected and analyzed to quantify the amounts of white blood cells (WBCs) and platelets (PLT) via a blood cell analyzer (Sysmex XE-2100, Japan), and simultaneously the serum was used to quantify the contents of aspartate aminotransferase (AST) and creatine kinase isoenzymes (CK-MB) via an automatic biochemical analyzer (Beckman Coulter AU2700, USA).

### Statistical analysis

GraphPad Prism software was used to make the graphs and statistically analyze the data. The data in the text and tables are showed as mean ± standard deviation (SD) whereas the data in graphs are showed as mean ± standard error of mean (SEM). Statistical analysis was performed using the paired two-tailed Student’s *t* test between two groups. P < 0.05 was considered a statistically significant difference.

## Results and discussion

### Isolation/purification of cell-bound membrane vesicles (CBMVs), drug loading, and verification/characterization

**Fig. 1:**
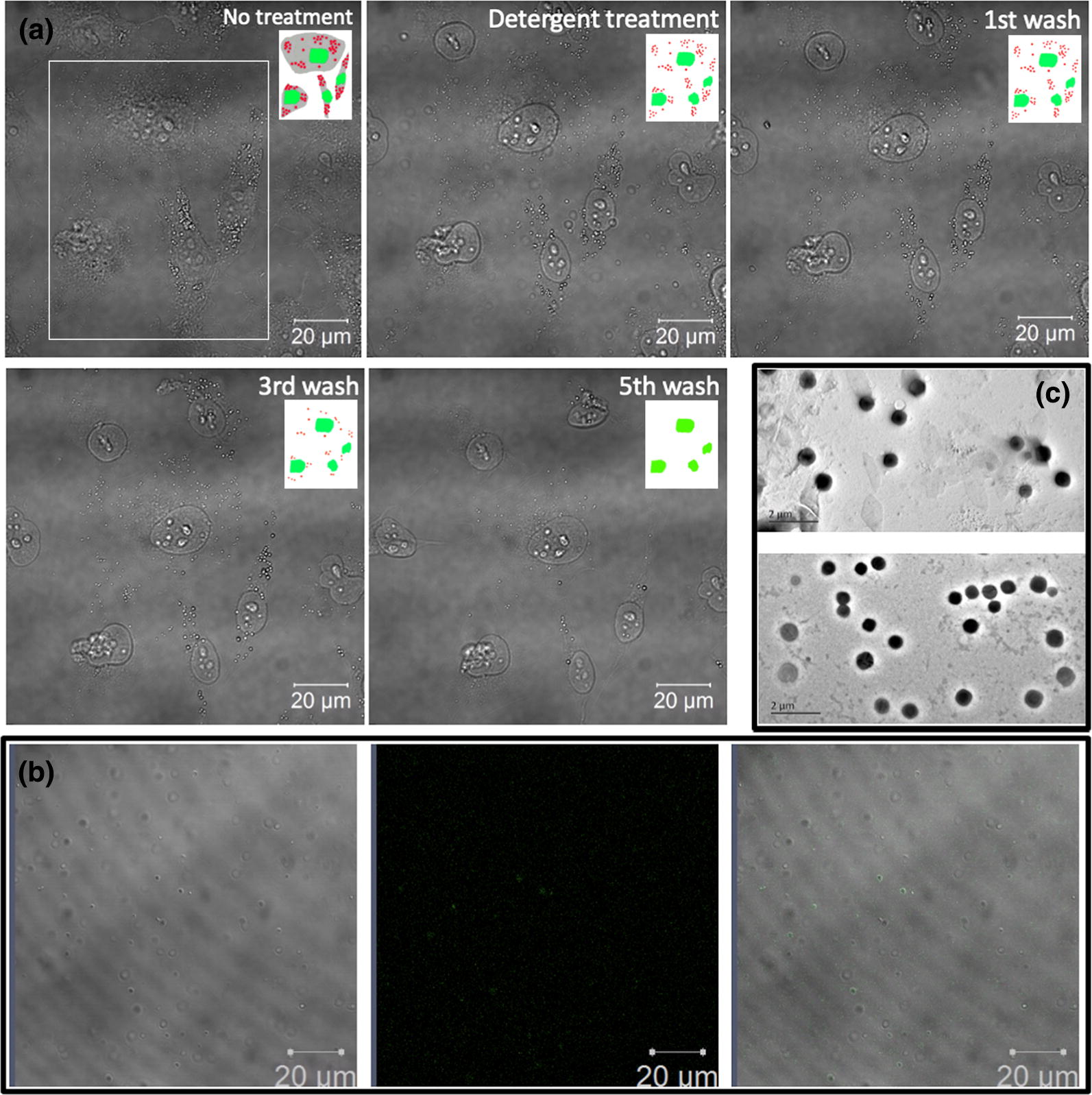
Isolation, doxorubicin loading, and morphology of cell-bound membrane vesicles (CBMVs). **a** Dynamic observation of the isolation process showing the same living endothelial cells (HUVECs) before and after detergent treatment (0.1% Triton X-100 at 37 °C for ~ 2–3 min) and after the 1st or 3rd or 5th PBS wash. Insets: schematic diagram showing the changes of the cells in the white box (red: CBMVs; green: nuclei; gray: cytoplasma). The disappearing red dots in the insets (i.e. isolated CBMVs) were collected for the subsequent drug loading. **b** Confocal microscopic images of isolated CBMVs loaded with doxorubicin (Dox-CBMVs; a movie was provided as Additional file 1: Movie S1 in the Additional file ). Left: differential interference contrast (DIC) image; middle: fluorescence image; right: merged image. **c** Transmission electron microscopic (TEM) images of isolated CBMVs (upper panel) and the Dox-CBMVs (lower panel)

Triton X-100, a relatively mild detergent, was utilized to isolate CBMVs from the cell surfaces. Due to the resistance to detergent, the CBMVs and nuclei of cells remained intact on the substrate whereas the other parts of cells were almost gone after Triton X-100 treatment (panels 1 and 2 of Fig. 1a; in the insets, the red, green, and gray represent CBMVs, nuclei, and the cytoplasma, respectively). Simple buffer replacements (or very gentle washes) for 1–3 times could remove the destroyed parts as well as potential extracellular vesicles generated transiently (panels 3 and 4 of Fig. 1a) and further gentle washes could collect the CBMVs leaving only the nuclei on the substrate (panel 5 of Fig. 1a). Centrifugation at a relatively low speed was performed to remove the nuclei potentially detached from the substrate. A further 10% sucrose density centrifugation at a high speed (200,000×*g* at 4 °C for 90 min) was conducted to purify the CBMVs.

Ultrasonication was used to help load the drug doxorubicin (Dox) and dialysis was utilized to remove the potentially excess free doxorubicin. High performance liquid chromatography (HPLC) revealed that the entrapment efficiency (EE) and drug loading efficiency (DL) of Dox-CBMVs were ~ 45% and ~ 2.4%, respectively (Table 1).

**Table 1:** Mean size, zeta potential, polydispersity index, entrapment efficiency (EE), and drug loading efficiency (DL) (mean ± SD, n = 3)

	Vesicles (CBMVs)	Dox-loaded vesicles (Dox-CBMVs)
Mean size (nm)	336.9 ± 5.1	395.9 ± 15.8
Zeta potential (mV)	−15.03 ± 0.38	−19.27 ± 1.44
Polydispersity index (PDI)	0.18 ± 0.03	0.46 ± 0.04
EE (%)	–	44.77 ± 2.71
DL (%)	–	2.36 ± 0.05

Then, confocal microscopy was recruited to confirm the successful Dox loading of CBMVs by imaging the co-localization of the isolated vesicles with the auto-fluorescent doxorubicin (Fig. 1b and Additional file 1: Movie S1 in the Additional file). Transmission electron microscopy observed the globular shape and nanometer size of Dox-CBMVs (lower panel of Fig. 1c) similar to those of Dox-free CBMVs (upper panel of Fig. 1c). Actually, dynamic laser scattering (DLS) analysis quantified that drug loading caused a slight increase in average size from ~ 337 nm for CBMVs to ~ 396 nm for Dox-CBMVs and in size distribution (i.e., polydispersity index) from ~ 0.18 for CBMVs to ~ 0.46 for Dox-CBMVs as well as in the absolute value of zeta potential from ~ 15 mV for CBMVs to ~ 19 mV for Dox-CBMVs (Table 1). The increases in vesicle size and size distribution indirectly reflect the successful loading of Dox while the increase in zeta potential implies that Dox-CBMVs were probably more stable and more resistant to aggregation than Dox-free CBMVs.

To exclude the possibility of CBMVs deriving from the components of the plasma membrane due to the Triton X-100 treatment, a plasma membrane fluorescent dye (Cellmask) was utilized to stain the plasma membrane of cells (Additional file 2: Figure S1 in the Additional file). If CBMVs were derived from the fluorescently stained plasma membrane, the fluorescence from CBMVs should be detected after Triton X-100 treatment. Before Triton X-100 treatment, the plasma membrane of the cells were stained in orange by Cellmask (the left and middle images of Additional file 2: Figure S1). After treatment, the fluorescence on cells disappeared due to the depletion of the plasma membrane by Triton X-100 whereas the CBMVs remained without fluorescence (the right image of Additional file 2: Figure S1), implying that CBMVs were not derived from the components of the plasma membrane.

### Dox-CBMVs have the cancer cell-killing ability by entering cell nuclei

**Fig. 2:**
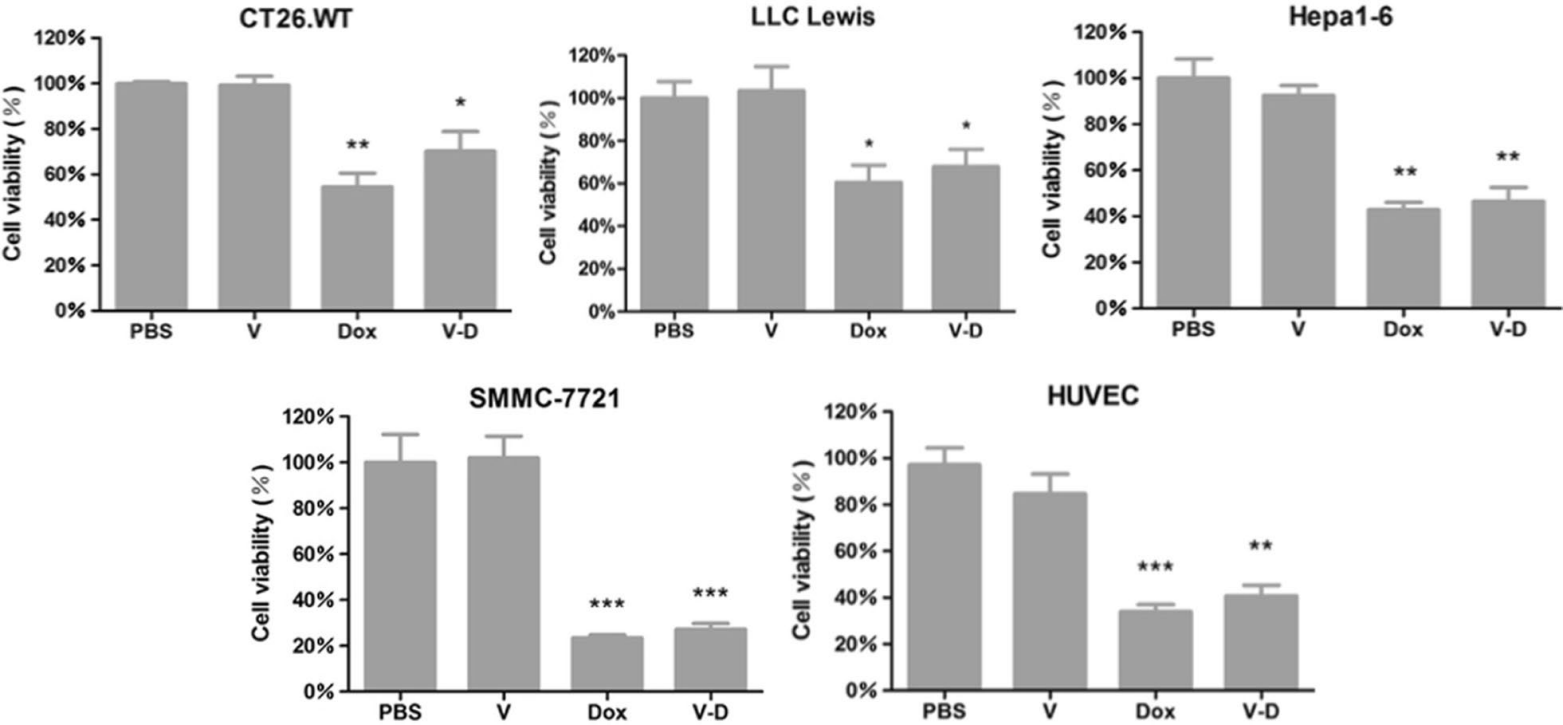
The cell-killing ability of isolated cell-bound membrane vesicles loaded with doxorubicin (Dox-CBMVs at the Dox concentration of 10 μg/mL; i.e. V–D in the graphs) detected by MTT assay. Three mouse cancer cell types (colon cancer CT26.WT cells, lung cancer LLC Lewis cells, and liver cancer Hepa1-6 cells), one human cancer cell types (liver cancer SMMC-7721 cells) and one human healthy cell type (HUVECs) were treated at 37 °C for 24 h. *, **, and *** represent p < 0.05, p < 0.01, and p < 0.001 compared with the PBS group or the group of CBMVs loaded without Dox (V in the graphs), respectively

Next, the drug efficacy at cellular level (i.e., the in vitro cancer cell-killing ability) of free Dox and Dox-CBMVs was evaluated via MTT assay after a treatment for 24 h (Fig. 2). The data shows that Dox-free CBMVs had no effects on cell viability whereas both free Dox and Dox-CBMVs significantly impaired the cell viability of all tested cell types including four cancer cell types (CT26.WT, LLC Lewis, Hepa1-6, and SMMC-7721 cells) and one healthy cell type (HUVECs). The data indicates that both free Dox and Dox-CBMVs had the cancer cell-killing ability but in a cell type-nonspecific manner. Moreover, the cell-killing ability of Dox-CBMVs was slightly, although not significantly, weaker than that of free Dox.

**Fig. 3:**
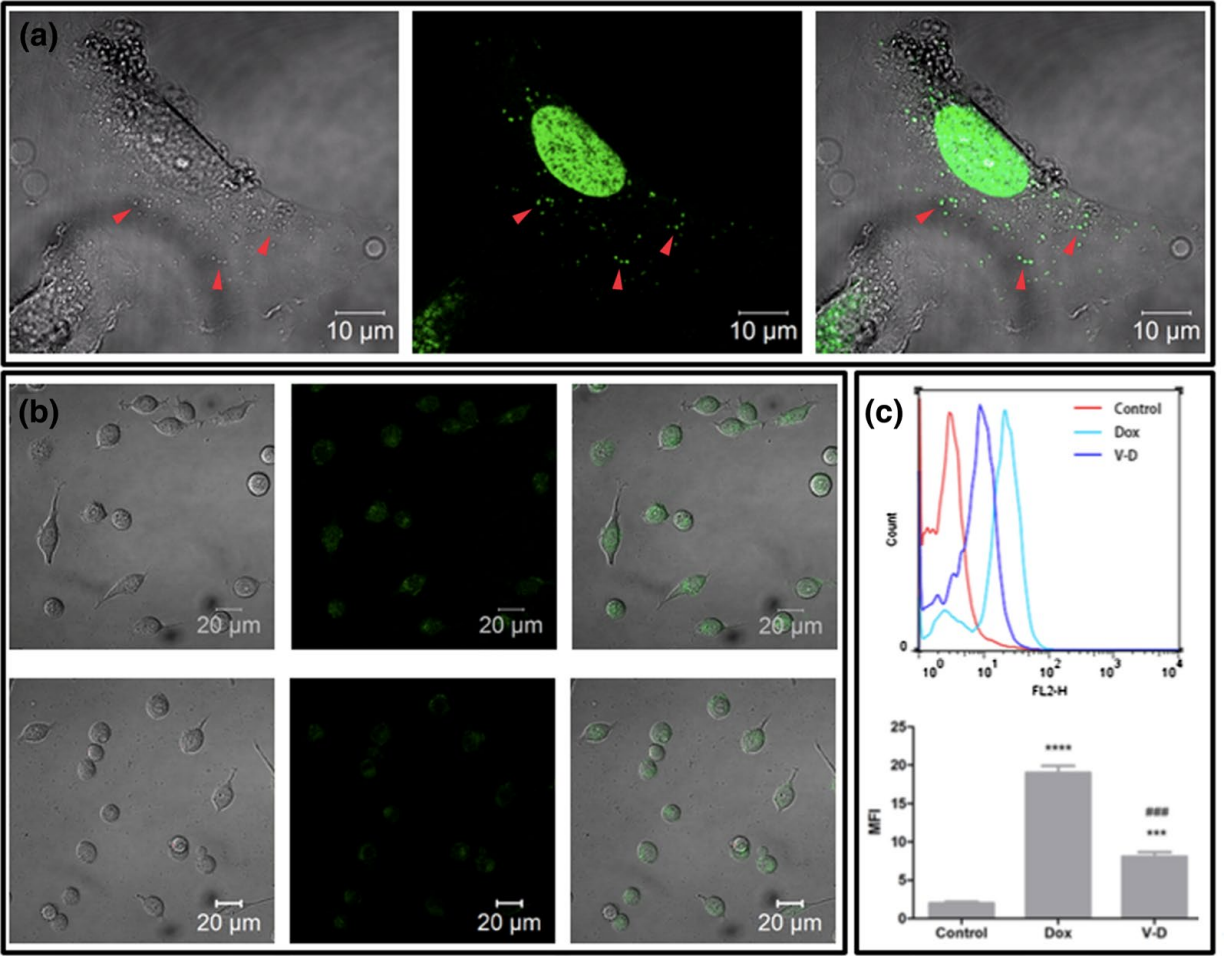
Cellular binding, internalization, and nucleus-targeting of isolated cell-bound membrane vesicles loaded with doxorubicin (Dox-CBMVs). **a** Confocal microscopic images of Dox-CBMVs (indicated by red arrowheads) binding onto the surfaces of endothelial cells (HUVECs). The cells were incubated with Dox-CBMVs at 37 °C for 1 h. **b** Confocal microscopic images of free Dox (upper panel) and Dox-CBMVs (lower panel) inside colon cancer cells (CT26.WT cells). **a, b** Left: DIC images; middle: fluorescence images; right: merged images. **c** Flow cytometry data of Dox-positive CT26.WT cells. Upper panel: representative; lower panel: statistical quantification of mean fluorescence intensity (MFI). *** represents p < 0.001 compared with the control group whereas ^###^ represents p < 0.001 compared with the free Dox group. In **b** and **c**, the cancer cells were incubated with free Dox or Dox-CBMVs (V–D in the graphs; at the Dox concentration of 10 μg/mL) at 37 °C for 2 h

The cellular binding and internalization abilities of CBMVs and/or Dox-CBMVs also were confirmed by confocal microscopy and flow cytometry (Fig. 3). After Dox-CBMVs were incubated with HUVECs for a short term (~ 1 h), many Dox-CBMVs (green dots indicated by the red arrowheads) still binding on cell surfaces could be observed although most Dox molecules had entered into cell nuclei (Fig. 3a). After free Dox or Dox-CBMVs were incubated with colon cancer cells (CT26.WT) for 2 h, fluorescent Dox molecules were observed in cell nuclei (Fig. 3b). The data confirm that Dox-CBMVs can be internalized by cells and that the cell-killing ability of Dox-CBMVs works by acting on the nucleus which is consistent with the mechanism of free Dox (i.e., intercalating with the DNA in the cellular nucleus) [22–24]. Further flow cytometric data displays that the mean fluorescence intensity (MFI) of CT26.WT cells treated by Dox-CBMVs was significantly lower than that of the cells treated by free Dox (Fig. 3c). It implies that the penetration of the Dox of Dox-CBMVs into cells was slower than that of free Dox. It also indirectly reflects the successful Dox loading of CBMVs and possibly explains the slightly weaker cell-killing ability of Dox-CBMVs than free Dox (Fig. 2).

**Fig. 4:**
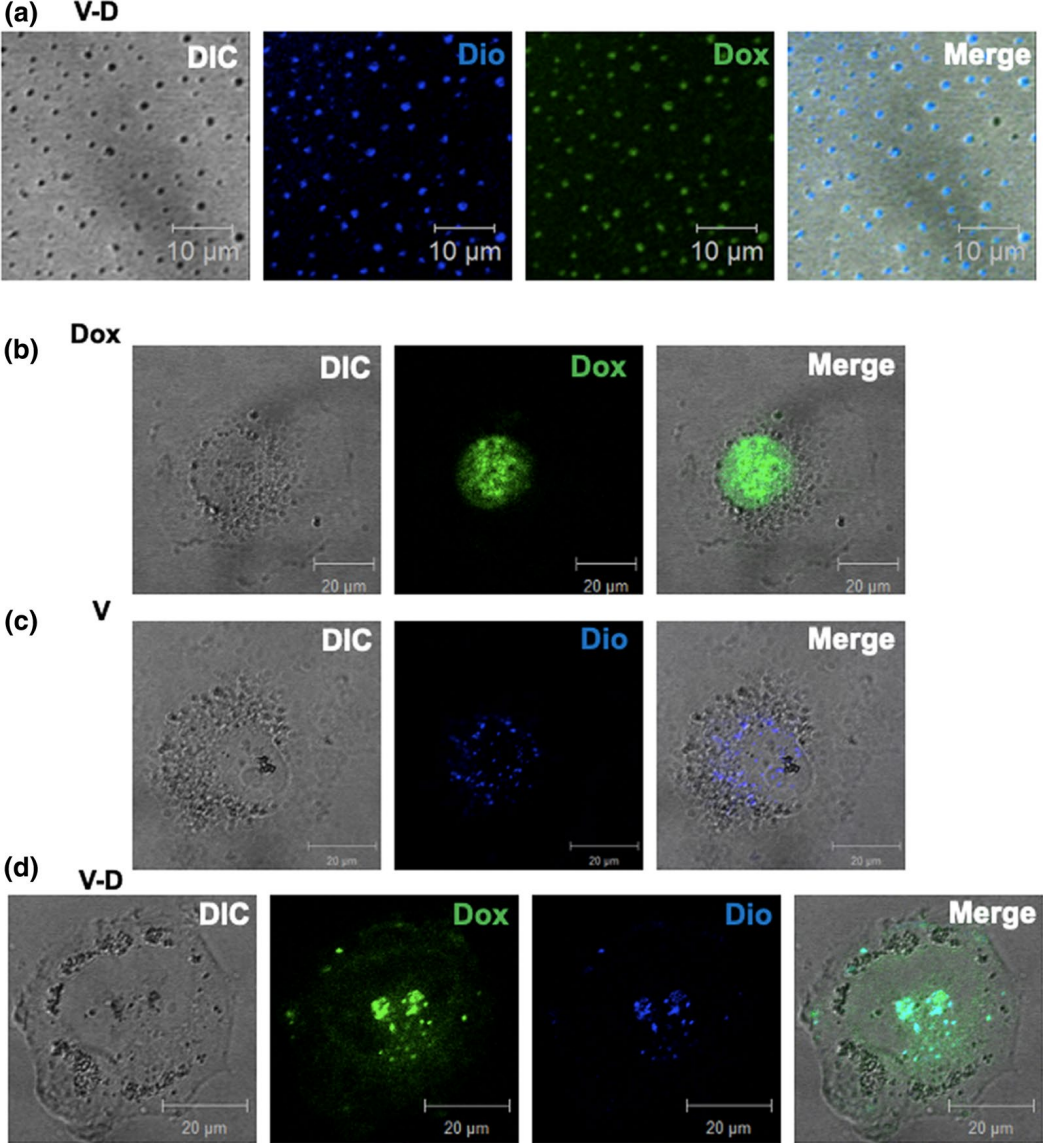
Co-localization of Dox and CBMVs in Dox-CBMVs and on cells. **a** Confocal microscopy detected the co-localization of Dox and CBMVs in Dox-CBMVs deposited on glass coverslips. The CBMVs were pre-stained with Dio (a cell membrane fluorescent dye; in blue, a pseudo color). The loaded Dox was self-fluorescent (in green). **b** Confocal microscopic images of free Dox in the nuclei of endothelial cells (HUVECs). **c** Confocal microscopic images of the Dio-stained CBMVs without Dox binding on the surfaces of HUVECs. **d** Confocal microscopic images of the Dio-stained Dox-CBMVs binding on the surfaces of HUVECs. The cells were incubated with free Dox, the Dio-stained CBMVs, and the Dio-stained Dox-CBMVs, respectively at 37 °C for 1 h

**Fig. 5:**
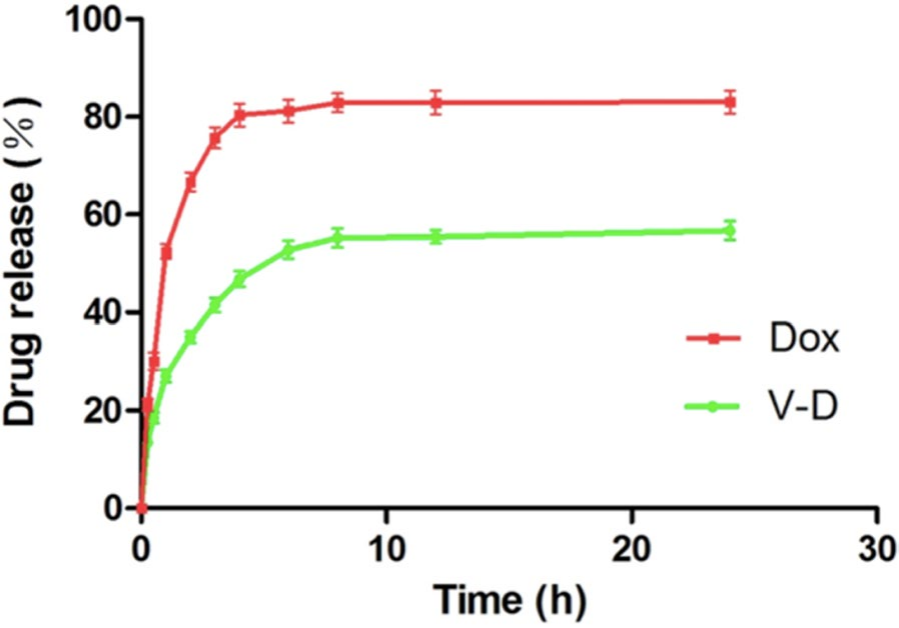
*In vitro* drug release profiles of doxorubicin from free doxorubicin (Dox) and isolated cell-bound membrane vesicles loaded with doxorubicin (Dox-CBMVs; V–D in the graph), respectively

To further confirm that the Dox molecules were really loaded in the CBMVs during internalization into cells, a cell membrane fluorescent dye Dio was used to stain the CBMVs therefore making it possible to detect the co-localization of Dox and CBMVs on/in cells. Figure 4a shows that the co-localization (the merged image) of Dox (green) and CBMV (blue) could be imaged in the Dox-CBMV particles (the DIC image) deposited on a glass coverslip, further confirming the successful Dox loading of CBMVs. Moreover, according to the images, it seems that almost all CBMV particles were loaded with Dox. After Dio-stained Dox-CBMVs were incubated with HUVECs for ~ 1 h, the co-localization of Dox and CBMV also was detected on the cells (Fig. 4b–d). The data implies that the Dox in CBMVs could enter cell nuclei via the internalization of Dox-CBMVs. However, it is hard to exclude the possibility that the free Dox released from Dox-CBMVs prior to the internalization of Dox-CBMVs (Fig. 5) could enter cell nuclei directly.

### Dox-CBMVs display a sustained drug release

Prior to the evaluation of drug efficacy at animal level, the in vitro drug release profiling was performed. Figure 5 shows that Dox-CBMVs had a slower drug release than free Dox (56.7 ± 2.8% and 83.0 ± 3.4% of total doxorubicin, respectively after 24 h). The data implies that Dox-CBMVs have a sustained drug release effect at least in vitro.

**Fig. 6:**
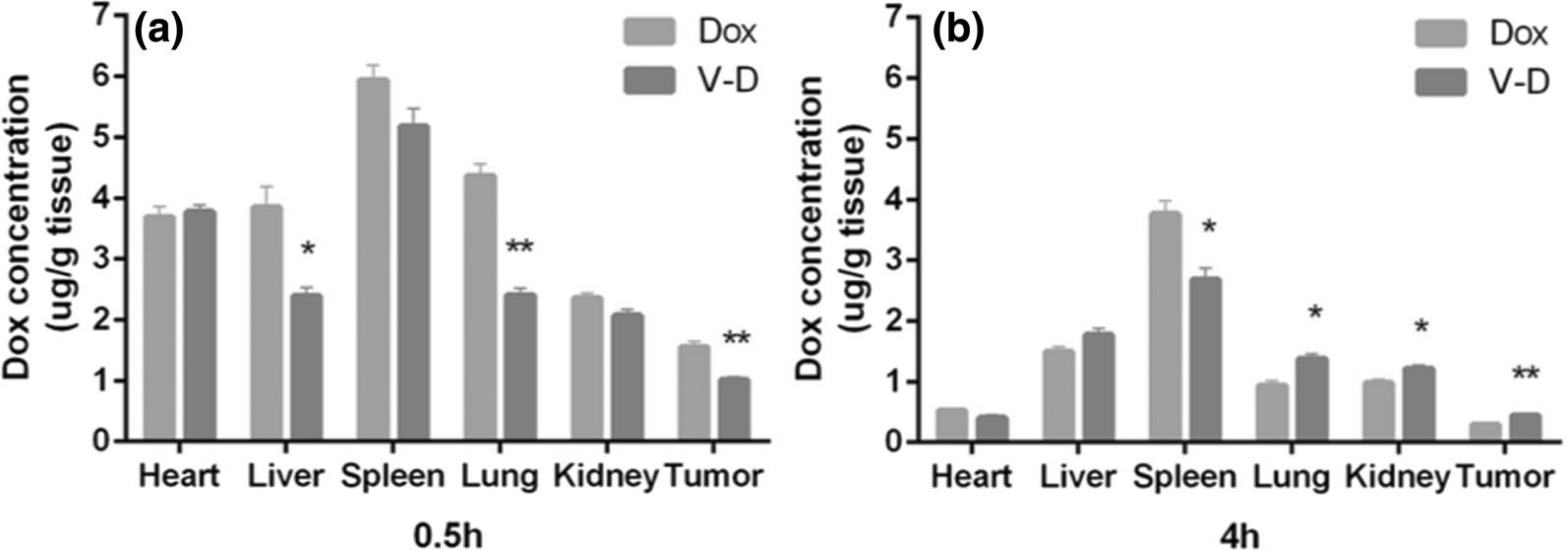
Tissue distribution of doxorubicin in colon tumor-bearing mice after a single drug administration. The tissue samples (heart, liver, spleen, lung, kidney, and tumor, respectively) were prepared at **a** 0.5 h and **b** 4 h after administration of free doxorubicin (Dox) or isolated cell-bound membrane vesicles loaded with doxorubicin (Dox-CBMVs; V-D in the graphs). * and ** represent p < 0.05 and p < 0.01 compared with the controls (the Dox groups), respectively

To determine whether Dox-CBMVs have tumor-targeting efficiency, the distribution of Dox in various tissues (heart, liver, spleen, lung, kidney, and tumor) of tumor-bearing mice at 0.5 h and 4 h was evaluated after a single drug administration (intravenous injection) of Dox-CBMVs or free Dox (Fig. 6 and Additional file 2: Figures S2–S3 in the Additional file). Take the colon tumor-bearing mice for example (Fig. 6), at both 0.5 h and 4 h after the *i.v.* administration, both free Dox and Dox-CBMV groups displayed the lowest Dox distribution in the tumor, implying that both free Dox and Dox-CBMVs have no tumor-targeting efficiency. On the other hand, at 0.5 h the Dox concentration in most tissues in the Dox-CBMV group was lower than that in the free Dox group (statistically significant differences occurred in the liver, the lung, and the tumor), implying that Dox-CBMVs are unable to promote the tumor targeting of Dox, but also implying that Dox-CBMVs may exert less side-effects on other tissues compared with free Dox. At 4 h and 24 h (Additional file 2: Figure S2 in the Additional file), however, the Dox concentration in the tumor in the Dox-CBMV group was higher than that in the free Dox group, partially implying that Dox-CBMVs have a sustained drug release effect in vivo. Similar results were found for the lung tumor-bearing mice (Additional file 2: Figure S3 in the Additional file).

### Dox-CBMVs have an efficient antitumor effect in vivo

**Fig. 7:**
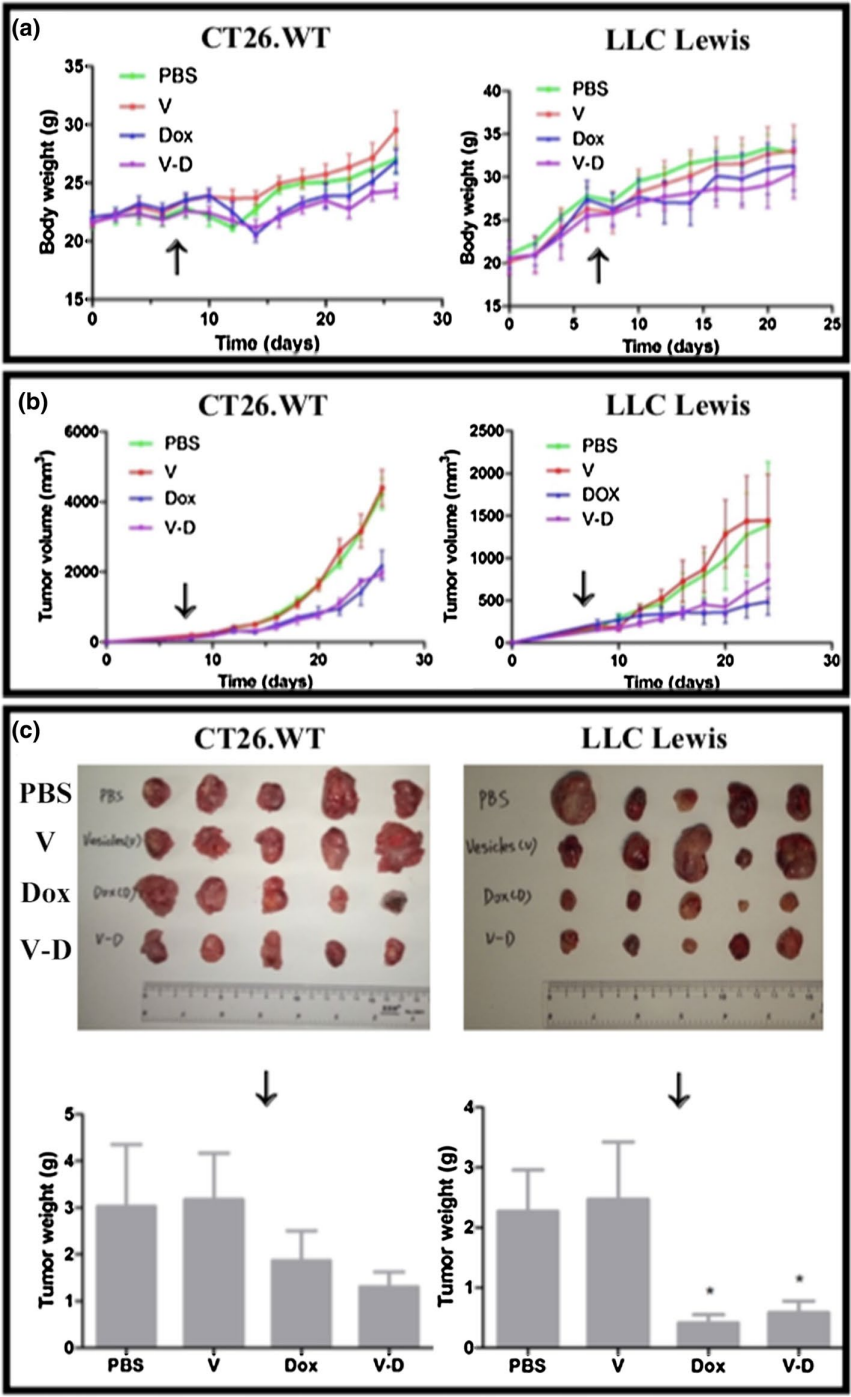
Antitumor effect of isolated cell-bound membrane vesicles loaded with doxorubicin (Dox-CBMVs). The dynamic changes in mouse body weight **a** and tumor volume **b** were measured every other day (The arrows in the graphs indicate the timepoint starting to administrate drugs). At the end of the experiments, the mice were imaged (see Additional file 2: Figure S4 in Additional file), and the tumors were excised (upper panel of **c**) and weighted (lower panel of **c**). CT26.WT cells (left panels) and LLC Lewis tumor blocks (right panels) were engrafted subcutaneously to establish mouse colon and lung tumor models, respectively

**Fig. 8:**
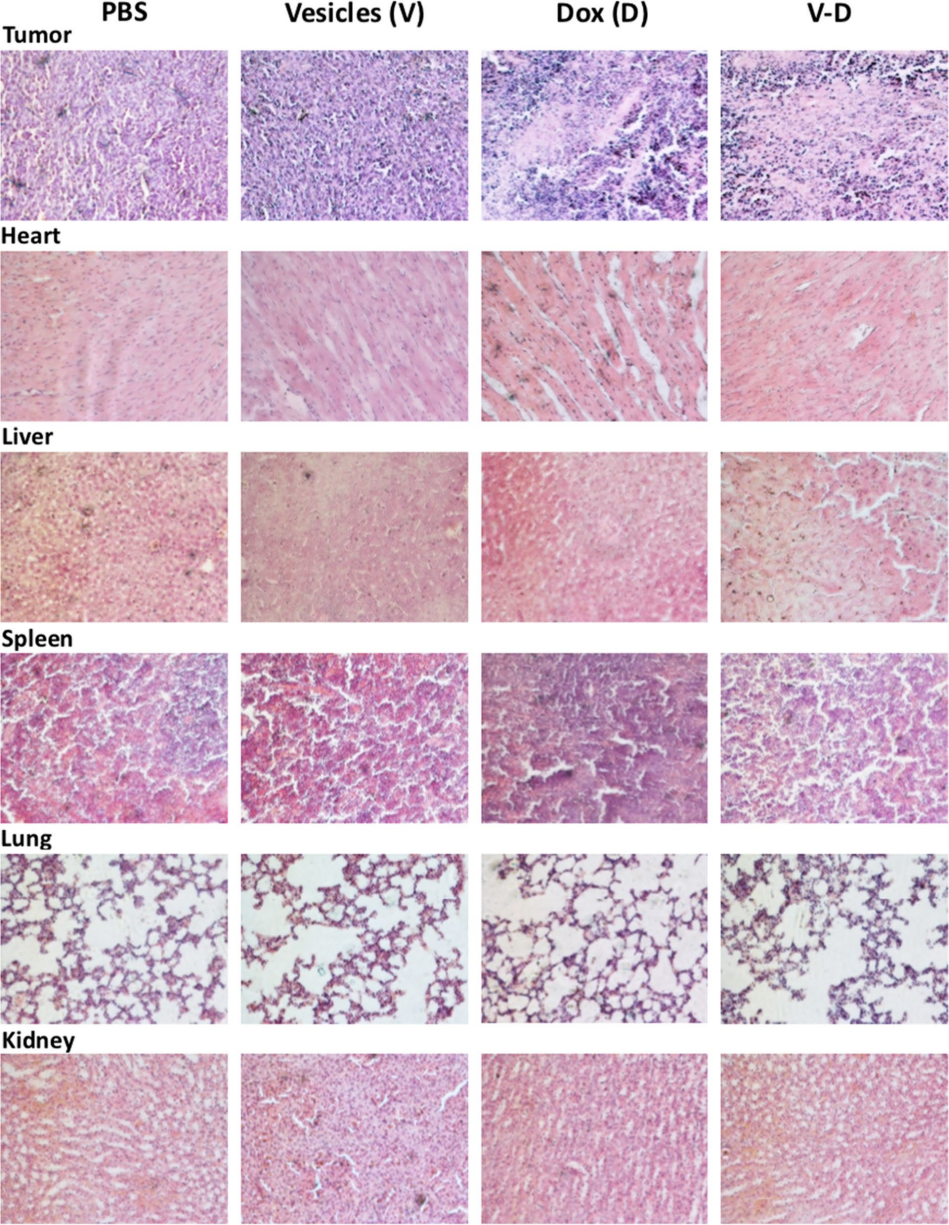
Histological analysis of colon tumor-bearing mice treated with isolated cell-bound membrane vesicles loaded with doxorubicin (Dox-CBMVs; or V–D) via H&E staining. Upper panel to bottom panel: tumor, heart, liver, spleen, lung, and kidney, respectively; left panel to right panel: PBS, CBMVs only, Dox only, and Dox-CBMVs, respectively

**Fig. 9:**
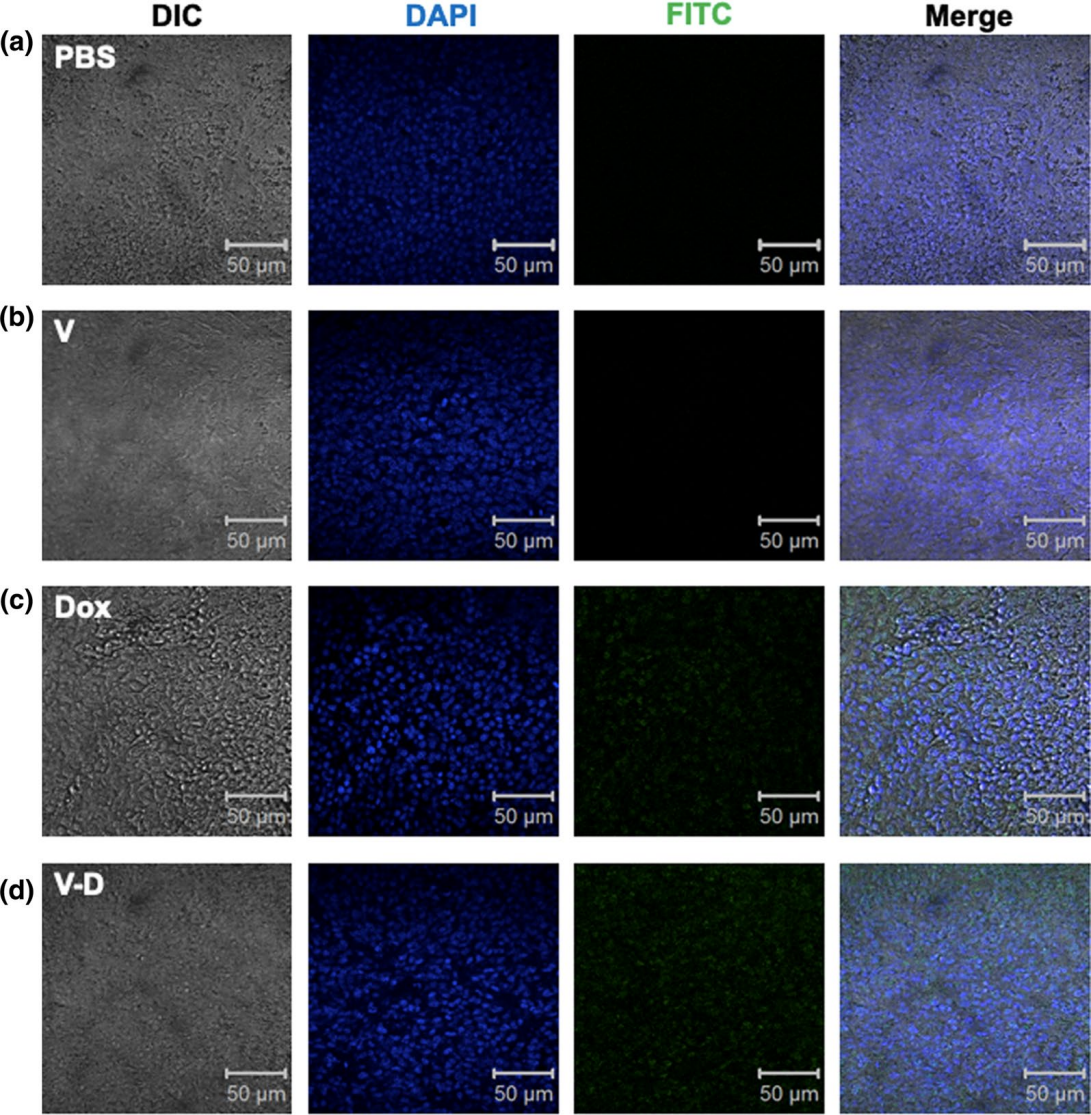
Apoptotic cell detection in tumor via TUNEL assay. The tissue slides of colon tumor were from the mice treated with PBS (**a**), empty CBMVs (**b**), free Dox (**c**), and Dox-CBMVs (**d**), respectively. Panels from left to right show DIC images, fluorescent images of cell nuclei (stained with DAPI; in blue), fluorescent images of the apoptotic cells (stained with TRITC; in green), and the merged images, respectively

Subsequently, the antitumor effect of Dox-CBMVs was tested at animal level (Fig. 7 and Additional file 2: Figure S4 in the Additional file). Mouse colon and lung tumor models were applied by subcutaneously engrafting colon cancer cells (CT26.WT) and small tumor blocks derived from lung cancer cells (LLC Lewis), respectively. The data show that the average tumor size/weight of mice treated with Dox-CBMVs was smaller/lighter than that of mice treated with PBS or Dox-free CBMVs but similar to that of mice treated with free Dox. It implies that Dox-CBMVs have an efficient antitumor effect but not better than free Dox probably due to their lack of tumor-targeting ability. Further histological analysis via H&E staining revealed that both free Dox and Dox-CBMVs inhibited the proliferation of cells in tumor (the upper panel of Fig. 8) but exerted no obvious damage on other tissues (the other panels of Fig. 8). The TUNEL assay (Fig. 9) also discovered that in comparison with the controls including PBS (Fig. 9a) and empty CBMVs (Fig. 9b) both Dox (Fig. 9c) and Dox-CBMVs (Fig. 9d) induced obvious cell apoptosis in the tumor. It confirms that Dox-CBMVs have an efficient antitumor effect but no histologically observed side-effects on other major organs.

The enhanced permeability and retention (EPR) effect might also contribute to the efficient antitumor effect of Dox-CBMVs in this study. Because of the leaky vasculature system (0.1–2 μm in diameter of endothelial gaps depending on the tumor size and type) and the poor lymphatic system in tumor tissues, nanoparticles could easily penetrate through the vessels and be detained inside a tumor for a relatively long term [25, 26]. The EPR effect might enhance the accumulation of Dox-CBMVs with a diameter of ~ 0.3–0.4 μm in tumor tissues. However, due to the lack of a tumor-targeting effect of Dox-CBMVs, the EPR effect probably was greatly limited. It is possible that the drug-loaded CBMVs with specific modifications for a good targetability to tumor will have greater antitumor efficacy due to both targetability and EPR effect.

### Dox-CBMVs exert less in vivo side effects compared with free dox

**Fig. 10:**
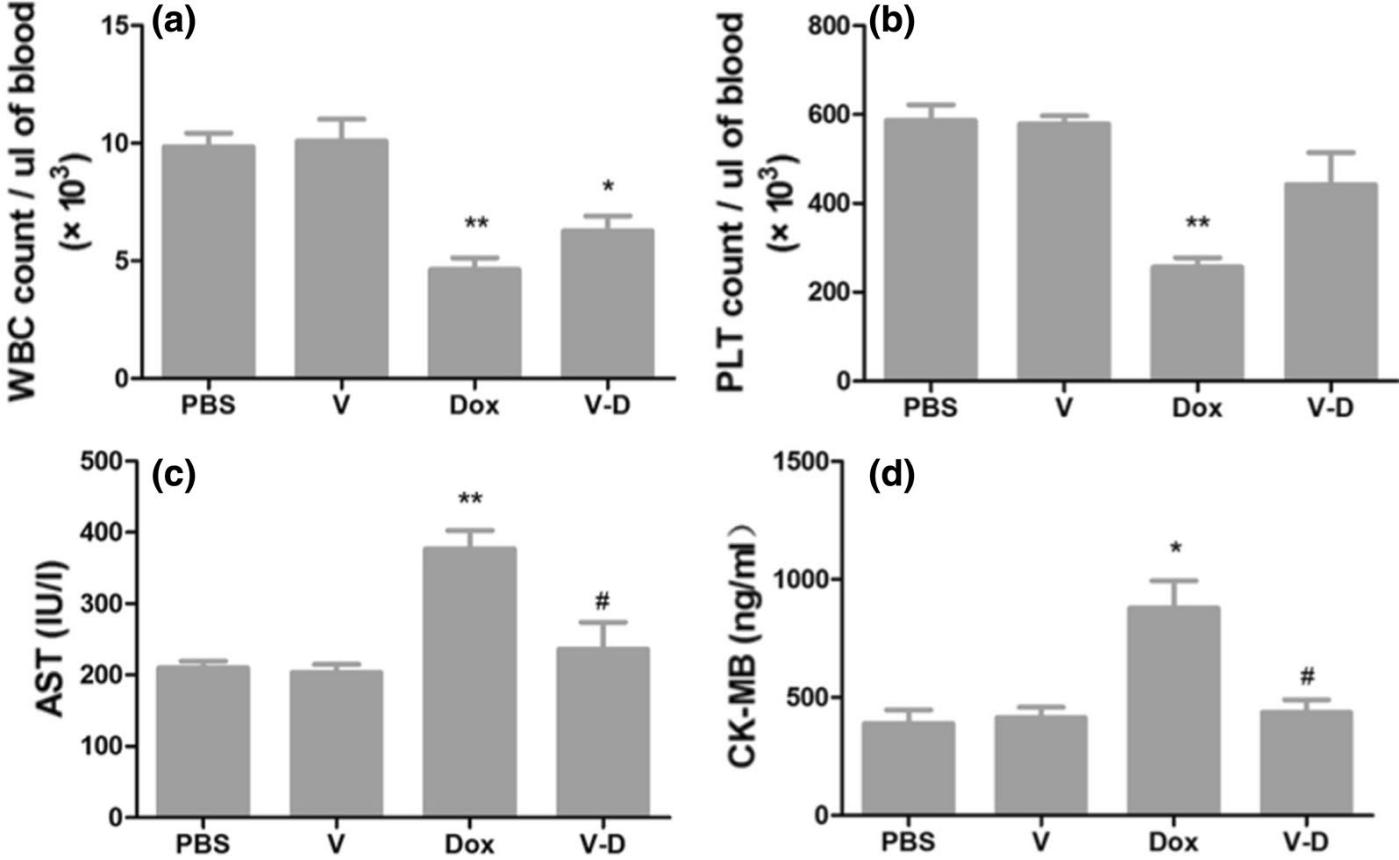
Evaluation of some potential side effects of free doxorubicin (Dox in the graphs) and isolated cell-bound membrane vesicles loaded with doxorubicin (Dox-CBMVs; V-D in the graphs). **a** The number of white blood cells (WBCs) in blood; **b** The number of platelets (PLT) in blood; **c** The concentration of aspartate aminotransferase (AST) in blood; **d** The concentration of creatine kinase isoenzymes (CK-MB) in blood. * and ** represent p < 0.05 and p < 0.01 compared with the controls, respectively; # represents p < 0.05 compared with the Dox group

Finally, several potential side effects of free Dox and Dox-CBMVs were evaluated (Fig. 10). It is well known that free Dox can cause multi-organ toxicities (particularly cardiotoxicity and bone marrow toxicity) [27, 28]. The decreases in amounts of circulating white blood cells (WBCs) and platelets (PLT) can reflect the Dox-induced myelosuppression whereas the increases in blood levels of creatine kinase isoenzymes (CK-MB) and aspartate aminotransferase (AST) can reflect the Dox-induced cardiac and liver injury. Our study confirms these side effects of free Dox (Fig. 10). Moreover, we found that Dox-CBMVs reversed the effects of free Dox recovering the amounts of WBCs and platelets (Fig. 10a, b) and the concentrations of AST and CK-MB (Fig. 10c, d) to the levels of the controls (the PBS group and the Dox-free vesicles group), implying that Dox-CBMVs probably can exert less side effects on other tissues. Multiple factors might contribute to the less side effects of Dox-CBMVs including the sustained drug release (Fig. 5) and the slower uptake by cells or tissues (Figs. 3 and 6).

## Conclusions

In recent decades, many biomembrane-wrapped drug delivery systems have been rapidly developed. One strategy is to reconstitute the natural biosystems by mimicking their basic components, such as the well-known liposomes [29, 30] and the reconstituted lipoproteins (e.g. reconstituted low/high-density lipoproteins) [20, 31, 32]. Another strategy is to directly recruit natural biosystems including virus/phage-based [33, 34], bacteria-based [35], and cell-based systems [36, 37] for drug delivery. Some cellular structures naturally released from cells (e.g. extracellular vesicles, particularly exosomes) have also been used as drug delivery systems [9, 38, 39]. To date, however, no cellular structures artificially isolated from cells have been developed as drug delivery systems.

For the first time, by using doxorubicin (Dox) as an antitumor drug model, this study provides evidence supporting the hypothesis that isolated nanometer-sized cell-bound membrane vesicles (CBMVs) can be developed to a novel class of drug delivery systems. Although it is currently unclear why and how Dox can be entrapped by CBMVs (perhaps similar to the drug entrapment by other membrane vesicles e.g., liposomes or extracellular vesicles) CBMVs probably can be used to deliver other antitumor drugs or even some drugs treating other diseases. This probability can be tested in the future. In the present study, as a drug delivery system the CBMVs derived from cultured endothelial cells (HUVECs) have disadvantages (e.g., currently no tumor targetability as a drug nanocarrier) and advantages (e.g., less side effects of the entrapped drug) as well as some potential advantages similar to other biocompatible, biodegradable delivery systems [24, 40]. Some specific modifications of CBMVs probably can be performed in the future to improve the tissue/tumor targetability of CBMVs. It also can be tested whether CBMVs derived from different cell types (e.g., cancer cells) have an enhanced tissue/tumor targetability. For the first time, the membrane vesicles at the cell surfaces (i.e., CBMVs) instead of extracellular vesicles are applied as a drug delivery system which may open a new door for the development of drug delivery systems.

## Supplementary information

**Additional file** **1****: Movie S1.**

**Additional file 2: Figure S1.** The cell-bound membrane vesicles (CBMVs) were not derived from the components of the plasma membrane depleted by Triton X-100. **Figure S2.** Tissue distribution of doxorubicin in colon tumor-bearing mice after a single drug administration for 24 h. **Figure S3.** Tissue distribution of doxorubicin in lung tumor-bearing mice after a single drug administration. **Figure S4.** Photos of the tumor-bearing mice with different treatments before the tumors were excised from the mice.

## Data Availability

All data generated or analyzed during this study are included in this article.

## Abbreviations

AST: Aspartate aminotransferase
CBMVs: Cell-bound membrane vesicles
CK-MB: Creatine kinase isoenzymes
DL: Drug loading efficiency
DLS: Dynamic light scattering
DMEM: Dulbecco’s Modified Eagle’s medium
Dox: Doxorubicin EE: entrapment efficiency
EVs: Extracellular vesicles
FBS: Fetal bovine serum
HPLC: High-performance liquid chromatography
HUVECs: Human umbilical vein endothelial cells
MTT: 3-(4,5-dimethyl-2-thiazolyl)-2,5-diphenyl-2-H-tetrazolium bromide
PBS: Phosphate-buffered saline
PDI: Polydispersity index
PLT: Platelets
WBCs: White blood cells
