# Supplementary material for: Isolated cell-bound membrane vesicles (CBMVs) as a novel class of drug nanocarriers
Source: J Nanobiotechnology. 2020 May 6;18:69. doi: 10.1186/s12951-020-00625-2 (PMC7204042; doi:10.1186/s12951-020-00625-2)
Supplement: Supplementary file 2 — Additional file 2: Figure S1. The cell-bound membrane vesicles (CBMVs) were not derived from the components of the plasma membrane depleted by Triton X-100. Figure S2. Tissue distribution of doxorubicin in colon tumor-bearing mice after a single drug administration for 24 h. Figure S3. Tissue distribution of doxorubicin in lung tumor-bearing mice after a single drug administration. Figure S4. Photos of the tumor-bearing mice with different treatments before the tumors were excised from the mice. [file 12951_2020_625_MOESM2_ESM.pdf]

Additional file

Isolated cell-bound membrane vesicles (CBMVs) as a novel class of drug nanocarriers

Yang Zhang [a, b], Yang Liu [a], Wendiao Zhang [a], Qisheng Tang [a], Yun Zhou [a], Yuanfang Li [a], Tong Rong [a], Huaying Wang [a], and Yong Chen [a, *]

[a] Jiangxi Key Laboratory for Microscale Interdisciplinary Study, Institute for Advanced Study, Nanchang University, Nanchang, Jiangxi 330031, P. R. China

[b] School of Materials Science and Engineering, Nanchang University, Nanchang, Jiangxi, 330031, P.R. China

*Corresponding author: Yong Chen

Mailing address: 999 Xuefu Ave., Honggutan District, Nanchang, Jiangxi 330031, P. R. China

Tel/Fax: 86-791-83969963

E-mail: dr_yongchen@hotmail.com or tychen@ncu.edu.cn

Short title: CBMVs as a novel drug nanocarrier

Additional file:

Additional file 1 : movie S1

Additional file 2: figures S1-S4

Additional figures:

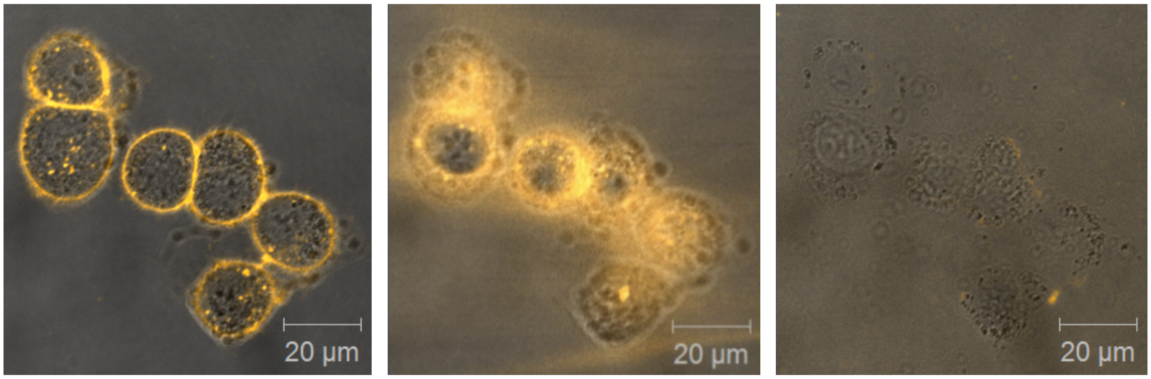

Fig. S1. The cell-bound membrane vesicles (CBMVs) were not derived from the components of the plasma membrane depleted by Triton X-100. The plasma membrane was stained by the Cellmask fluorescent dye (CellMask Orange plasma membrane stain, ThermoFisher Scientific, NY) prior to Triton X-100 treatment. (Left) Confocal imaging of the HUVEC cells before Triton X-100 treatment. (Middle) The same cells before treatment but on different focal plane. (Right) The same cells after 0.1% Triton X-100 treatment. The showed images are merged from the DIC images and the fluorescent images. The images show that Triton X-100 treatment depleted the fluorescently-stained plasma membrane whereas the remaining CBMVs on the substrate did not display the fluorescence, implying that CBMVs were not derived from the components of the plasma membrane depleted by Triton X-100.

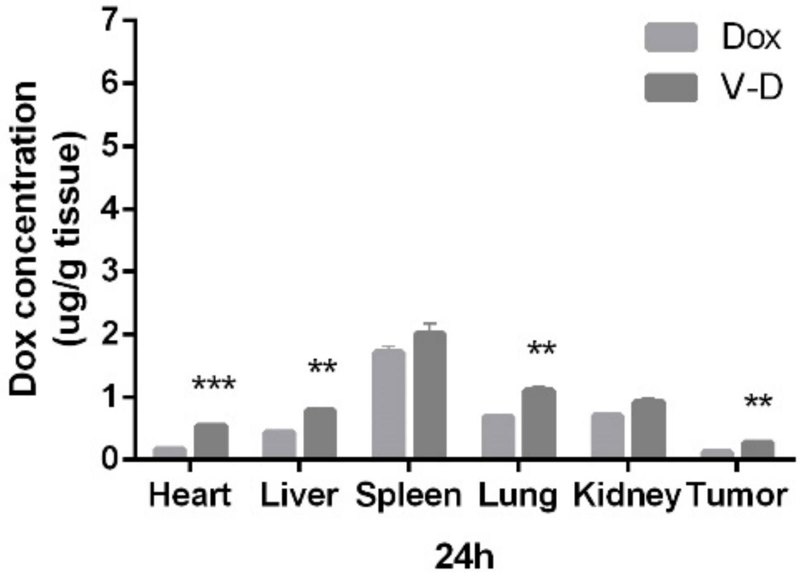

Fig. S2. Tissue distribution of doxorubicin in colon tumor-bearing mice after a single drug administration for 24 h. The tissue samples (heart, liver, spleen, lung, kidney, and tumor, respectively) were prepared at 24 h after administration of free doxorubicin (Dox) or isolated cell-bound membrane vesicles loaded with doxorubicin (Dox-CBMVs; V-D in the graphs). ** and *** represent $p<0.01$ and $p<0.001$ compared with the controls (the Dox groups), respectively.

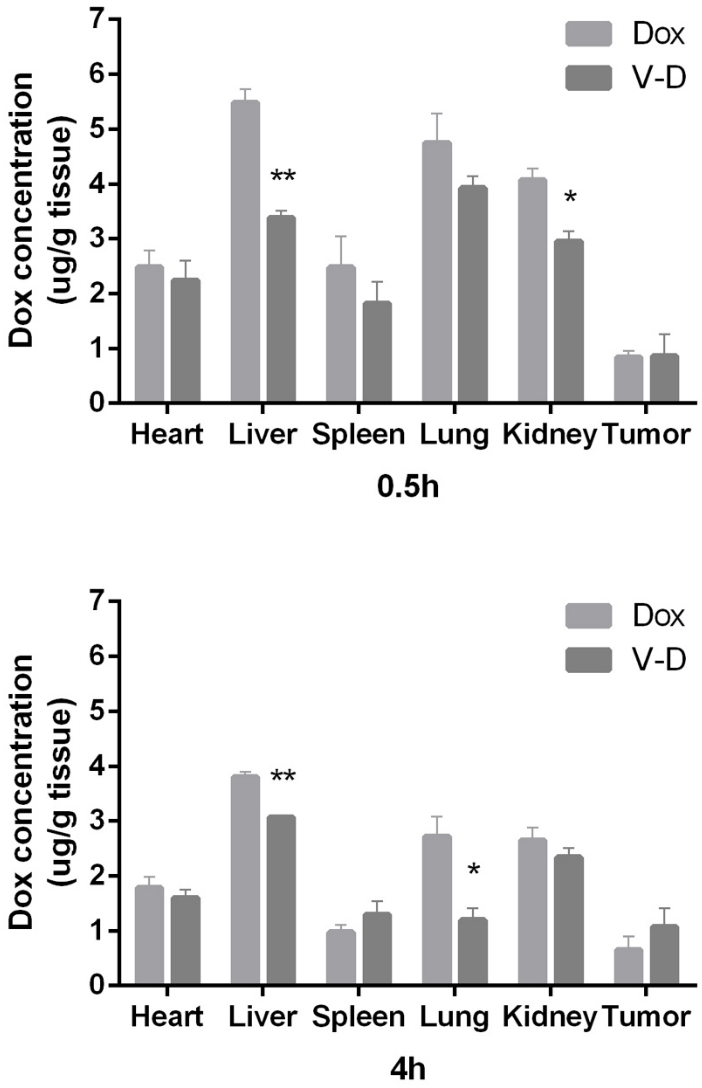

Fig. S3. Tissue distribution of doxorubicin in lung tumor-bearing mice after a single drug administration. The tissue samples (heart, liver, spleen, lung, kidney, and tumor, respectively) were prepared at 0.5 h (upper panel) and 4 h (bottom panel) after administration of free doxorubicin (Dox) or isolated cell-bound membrane vesicles loaded with doxorubicin (Dox-CBMVs; V-D in the graphs). * and ** represent $p<0.05$ and $p<0.01$ compared with the controls (the Dox groups), respectively.

models, respectively.

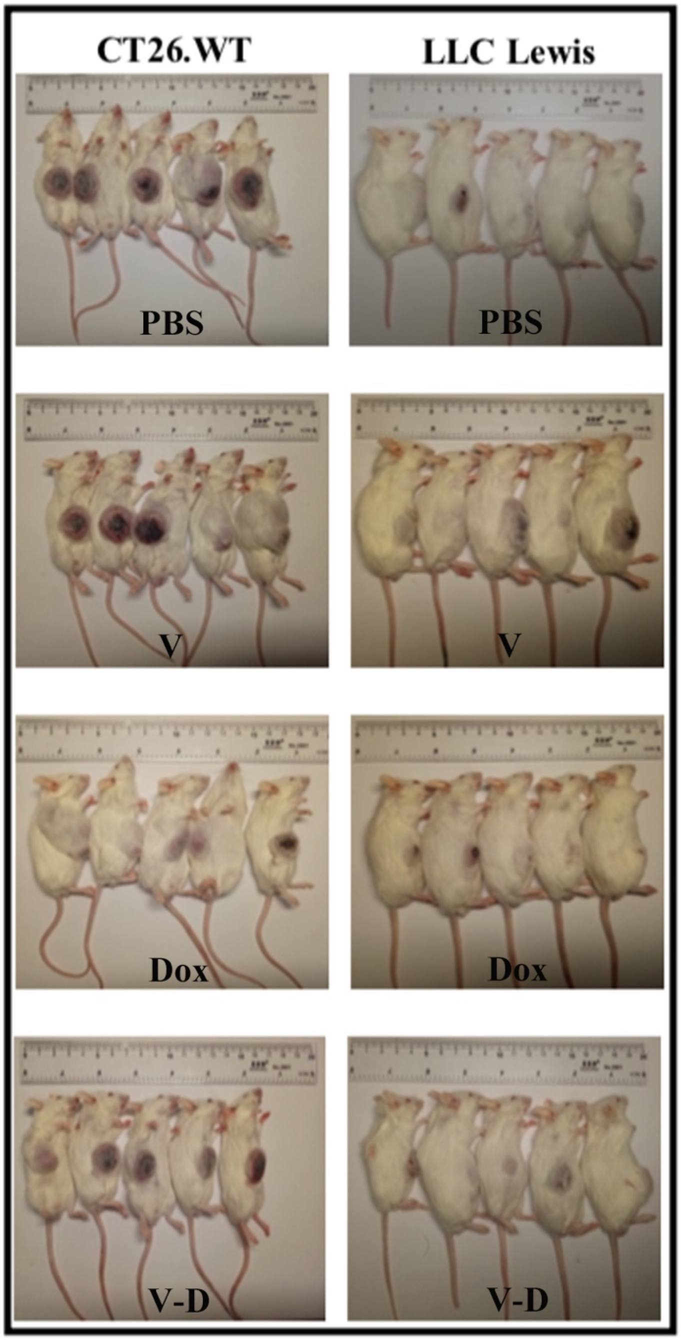

Fig. S4. Photos of the tumor-bearing mice with different treatments before the tumors were excised from the mice. CT26.WT cells (left panels) and LLC Lewis tumor blocks (right panels) were engrafted subcutaneously to establish mouse colon and lung tumor models, respectively.
